# Evaluation of the susceptibility to fipronil of *Rhipicephalus microplus* larvae from egg masses incubated at different times of oviposition

**DOI:** 10.29374/2527-2179.bjvm005922

**Published:** 2023-03-22

**Authors:** Daniela de Oliveira Rocha, Roxanne Marina da Silva Roque, Thiago de Souza Vieira, Ingrid Lins Raquel de Jesus, Brena Gava Guimarães, Marisa Beatriz da Silva Rocha, Fabio Barbour Scott, Barbara Rauta Avelar

**Affiliations:** 1 1- Undergraduate in Veterinary Medicine, Instituto de Veterinária (IV), Universidade Federal Rural do Rio de Janeiro. Seropédica, RJ, Brazil.; 2 2- Veterinarian, Resident. Programa de Residência em Medicina Veterinária - Diagnóstico em Parasitologia Animal, IV, UFRRJ. Seropédica, RJ, Brazil; 3 3- Undergraduate in Zootechnics, Instituto de Zootecnia (IZ), UFRRJ. Seropédica, RJ, Brazil.; 4 4- Pharmacist, Programa de Pós-Graduação em Ciências Veterinárias (PPGCV), Departamento de Parasitologia Animal (DPA), IV, UFRRJ. Seropédica, RJ. Brazil.; 5 5- Veterinarian, MSc. PPGCV, DPA, IV, UFRRJ, Seropédica, RJ. Brazil.; 6 Veterinarian, DSc. Departamento de Parasitologia Animal (DPA), Instituto de Veterinária (IV), Universidade Federal Rural do Rio de Janeiro (UFRRJ). Seropédica, RJ, Brazil.; 7 Veterinarian, DSc. PPGCV, DPA. IV, UFRRJ. Seropédica, RJ. Brazil.

**Keywords:** *in vitro* test, laboratory colonies, tick, teste *in vitro*, colônia laboratorial, carrapato

## Abstract

The objective of this work was to evaluate the susceptibility of *R. microplus* larvae from different oviposition times to fipronil. The LPT was performed in sextuplicate, at concentrations of 18.75, 37.5, 75, 150 and 300 µg.mL^-1^. The LC_50_ found for the egg masses incubated with +7, +14 and +21 days were respectively 105.87, 110.71 and 121.22 µg.mL^-1^. The larvae originating from egg masses from the same group of engorged females, incubated on different days, presented similar mortality rates compared to the evaluated fipronil concentrations, facilitating the maintenance of laboratory colonies of this tick species.

## Resumo

O objetivo deste trabalho foi avaliar a susceptibilidade de larvas de *R. microplus* oriundas de diferentes momentos da oviposição frente ao fripronil. O TPL foi realizado em sextuplicata, nas seguintes concentrações 18,75; 37,5; 75; 150; 300 µg.mL^-1^. Não houve diferença estatística entre as mortalidades das larvas oriundas de posturas incubadas nos dias mais sete, +14 e +21, expostas ao fipronil. As CL_50_ encontradas para as posturas incubadas com +7, +14 e +21 dias foram respectivamente 105,87; 110,71 e 121,22 µg.mL^-1^. Observou-se que as larvas oriundas de posturas, do mesmo grupo de fêmeas ingurgitadas, incubadas em dias diferentes apresentam taxas de mortalidade parecidas frente as concentrações de fipronil avaliadas, facilitando a manutenção das colônias laboratorias desta espécie de carrapato.

The tick *Rhipicephalus microplus* is the most important ectoparasite of cattle from an economic standpoint (Grisi et al., 2014). The control of this species is basically performed with synthetic acaricides, whose indiscriminate use leads to the emergence of resistant strains (Castro-Janer et al., 2009; Reck et al., 2014).

*In vitro* tests such as the larval packet test (LPT) are indicated to identify these strains. This and other *in vitro* methods are also used in the search for new compounds for the control of *R. microplus*. Laboratory colonies of this species are used by several researchers to carry out these tests, because they have known susceptibility to synthetic acaricides and do not suffer seasonal interference of the non-parasitic phase, because they are kept in incubators (Pereira et al., 2008; Castro-Janer et al., 2009; Reck et al., 2014; Figueiredo et al., 2018).

The maximum oviposition time described by Davey et al. (1980) is 21 days. It is customary for the maintenance of colonies to analyze samples of egg masses laid 21 days after the incubation of the engorged females. The eggs laid first hatched first, and a sample of egg masses weighed 21 days after female incubation contained larvae of different ages, whit have different susceptibilities to acaricides (Stone & Haydock, 1962). Thus, the objective of this work was to evaluate the susceptibility to fipronil of *R. microplus* larvae originating from different oviposition periods.

For this, all the egg masses of the same group of *R. microplus* females (100 ticks) were sampled at three moments counted from the start of oviposition (+7, +14 and +21 days), with every 0.5g of eggs placed in adapted 5mL syringes. Forty-two days after the onset of laying (after 21 days of incubation of the last group of egg masses), an *in vitro* test with fipronil was performed with larvae from egg mass samples collected at different times.

The tests were performed in sextuplicate at the Laboratory of Experimental Chemotherapy in Veterinary Parasitology of UFRRJ, with unfed larvae obtained from a laboratory colony (CEUA –number 4667181218). The LPT (Food and Agriculture Organization of the United Nations, 2004) was carried out with technical fipronil diluted in a solution prepared with one part of olive oil to two parts of trichloroethylene in the concentrations 18.75, 37.5, 75, 150 and 300 µg.mL^-1^. Filter papers measuring 8.5 x 7.5cm were impregnated with 670 µl of the concentrations of fipronil and as a negative control only the diluent was used. After two hours for evaporation of solvents from the filter paper, approximately 100 larvae were placed in packets formed by folding the filter paper sheets. These were closed with binder clips and incubated (27 ± 1 °C and 80 ± 10% relative humidity) for 24 hours for mortality assessment (% mortality = (total number of dead larvae x100) /total number larvae). Probit analysis was performed with the R 3.6.1 program and statistical analysis was carried out with the BioEstat 5.0 program. The data were transformed into Log 10 and the mean analysis was performed by ANOVA, with a confidence interval of 95%.

The mortality percentages are found in Table 1 and the probit analysis results in Table 2. The mortalities observed at the different concentrations were similar for the three groups, as well as the LC_50_ found.

**Table 1:** Mortality percentage of *Rhipicephalus microplus* larvae exposed to fipronil.

Concentration (µg.mL^-1^)	Percentage of mortality (%) of larvae from postures incubated at different times.											
	+ 7				+ 14				+ 21			
Control	0.55	±	0.74	^a^	6.05	±	2.28	^b^	2.06	±	3.11	^ab^
18.75	2.33	±	3.41	^a^	10.00	±	7.09	^b^	7.32	±	2.65	^ab^
37.5	13.62	±	12.68	^a^	19.85	±	8.01	^a^	14.53	±	26.89	^a^
75	32.86	±	31.53	^a^	36.78	±	41.66	^a^	47.43	±	39.14	^a^
150	70.05	±	30.96	^a^	50.13	±	25.49	^a^	46.74	±	31.00	^a^
300	85.45	±	3.36	^a^	86.61	±	5.15	^a^	79.36	±	11.96	^a^

ab different letters on the same row have statistical difference between means (p≤0.05).

**Table 2:** Values of LC_50_, slope, R^2^ and chi-square (X^2^) and *p* value of X^2^ of *Rhipicephalus microplus* larvae from postures incubated at different times of oviposition, exposed to fipronil.

Time of incubation of the egg mass	Median lethal concentration (LC_50_) (µg.mL^-1^)	Slope	R^2^	X^2^	*p*
+7	105.87 (99.78 – 112.37)	2.52 ± 0.13	0.95	13.36	0.99
+14	110.71 (102.05 – 120.57)	1.85 ± 0.24	0.98	59.04	1.00
+21	121.22 (111.88 – 131.78)	1.79 ± 0.29	0.94	95.09	1.00

The egg masses incubated +7 days after the beginning of laying by the females, as well as the those incubated at +14, started hatching on days close to the masses incubated on day +21, around 28 days after the start of laying, that is the period of egg development was not influenced by the order of oviposition (Hitchcock, 1955). The eggs at the end of laying underwent maturation while still inside the female, since the ovaries contain eggs at all stages of development when oviposition begins (Pereira et al., 2008).

Despite the significant variations in the mortality of the control groups, these were within the expected range for the LPT (Food and Agriculture Organization of the United Nations, 2004). These results are important for *R. microplus* colonies, since no alterations were observed in mortalities and LCs_50_ values, facilitating their maintenance, in which egg masses can be weighed in the same aliquot on day +21 after incubation of females, taking advantage of 100% of the oviposition period at 27 ± 1°C and 80 ± 10 relative humidity (Davey et al., 1980), without interfering with the results obtained in *in vitro* assays to diagnose resistance or to study new acaricidal compounds. The larvae from egg masses incubated on different days showed similar mortality rates when submitted to the evaluated fipronil concentrations.
